# Evaluation of Differences in Automated QT/QTc Measurements between Fukuda Denshi and Nihon Koden Systems

**DOI:** 10.1371/journal.pone.0106947

**Published:** 2014-09-17

**Authors:** Motoaki Sano, Yoshiyasu Aizawa, Yoshinori Katsumata, Nobuhiro Nishiyama, Seiji Takatsuki, Shigeo Kamitsuji, Naoyuki Kamatani, Keiichi Fukuda

**Affiliations:** 1 Department of Cardiology, Keio University School of Medicine, Tokyo, Japan; 2 StaGen Co. Ltd., Tokyo, Japan; University of Minnesota, United States of America

## Abstract

**Background:**

Automatic measurement becomes a preference, and indeed a necessity, when analyzing 1000 s of ECGs in the setting of either drug-inducing QT prolongation screening or genome-wide association studies of QT interval. The problem is that individual manufacturers apply different computerized algorithms to measure QT interval. We conducted a comparative study to assess the outcomes with different automated measurements of QT interval between ECG machine manufacturers and validated the related heart rate correction methods.

**Methods and Results:**

Herein, we directly compared these different commercial systems using 10,529 Fukuda Denshi ECGs and 72,754 Nihon Kohden ECGs taken in healthy Japanese volunteers. Log-transformed data revealed an equal optimal heart rate correction formula of QT interval for Fukuda Denshi and Nihon Kohden, in the form of QTc = QT/RR^−0.347^. However, with the raw data, the optimal heart rate correction formula of QT interval was in the form of QTc = QT+0.156×(1-RR) for Fukuda Denshi and QTc = QT+0.152×(1-RR) for Nihon Kohden. After optimization of heart rate correction of QT interval by the linear regression model using either log-transformed data or raw data, QTc interval was ∼10 ms longer in Nihon Kohden ECGs than in those recorded on Fukuda Denshi machines. Indeed, regression analysis revealed that differences in the ECG machine used had up to a two-fold larger impact on QT variation than gender difference. Such an impact is likely to be of considerable importance when ECGs for a given individual are recorded on different machines in the setting of multi-institutional joint research.

**Conclusions:**

We recommend that ECG machines of the same manufacturer should be used to measure QT and RR intervals in the setting of multi-institutional joint research. It is desirable to unify the computer algorithm for automatic QT and RR measurements from an ECG.

## Introduction

Prolongation of the QT interval is an intermediate phenotype associated with an individual’s increased propensity to develop a ventricular tachyarrhythmia called Torsades de Pointes and increased risk of sudden cardiac death (SCD) [1], [2], [3], [4].

All new drugs must undergo a 'thorough QT/QTc' (TQT) study to detect drug-induced QT prolongation. Indeed, in accordance with the GCP (good clinical practice) E14 guidance, development of several drugs that prolong QTc by >5 ms was abandoned by the relevant companies [5]. Therefore, accurate and consistent measurement of the QT/QTc interval is increasingly important not only for clinical benefit, but also from the pharmaceutical drug safety screening perspective.

It is well established that the rare Mendelian diseases of extreme QT duration, the long-QT syndromes [6] [7], are risk factors for SCD [8]. However, recent population-based cohort studies demonstrated that multiple common genetic variants that contribute to the repolarization affect the risk of SCD in the general population [9] [10]. Although individual common variants would be expected to induce only modest increments in QT interval (3–6 ms per locus) [10], these common variants could, individually and in aggregate, more markedly increase SCD through influencing susceptibility to arrhythmogenic triggers such as myocardial ischemia, electrolyte disturbance, or QT-prolonging medications [11]. In fact, a 10-ms increase in the observed QT interval was associated with an increased risk of SCD (hazard ratio, 1.19; 95% CI, 1.07 to 1.32; *P* = 0.002) [12].

QT interval is inversely correlated with heart rate. Generally, QT intervals are corrected for heart rate so that QTc is equal to QT if the heart rate is 60 beats per minute, i.e., RR interval of 1 s. Various formulae have been used to correct QT interval (QTc) with respect to heart rate, and such correction can be done by the linear regression model using either raw data or log-transformed data.

Measurement of QT interval by surface electrocardiogram (ECG) is performed either manually or automatically. Manual measurement, in which the end of T wave is determined as the intersection between the tangent to the steepest down-slope of the T wave and the isoelectric line, is made according to published guidelines, but is time-consuming and contains inter-reader variability. For automated measurements, difficulties in delineating the end of T wave are encountered when either the ECG is polluted with noise or the T wave is flat, bifid, biphasic, or overlapping on a U wave [13] [14]. However, automated QT measurement technologies are evolving rapidly and their precision has been increasingly demonstrated to be appropriate in various studies [15]. Automatic measurements also show greater consistency and are more suitable for processing a large volume of data.

In Japan, Nihon Kohden and Fukuda Denshi together provide most of the ECG machines used clinically. However, each of these manufacturers uses a different automatic computerized algorithm to measure QT interval and different methods for heart rate correction of QT interval, with Nihon Kohden adopting the ECAPS12 formula [QTc (ECAPs12) = QT+(1–RR)/7] and Fukuda Denshi the Bazett formula [QTc (Bazzet) = QT/RR^0.5^]. Surprisingly, the impact of such system differences on the clinical results has been ignored. Thus, we conducted a comparative study to assess the outcomes with different automated measurements of QT interval between ECG machine manufacturers and validated the related heart rate correction methods.

## Methods

### Ethics statement

Since all the data used in this study were anonymous data, the ethical committee of Fukuda Denshi and Nihon Kohden both had a same judgment that there is no ethical problem to provide us with anonymous data. IRB committee at Keio University said that the submission of this study for the approval by IRB is not necessary.

### Evaluation of automatic QT and RR measurements in adult resting ECGs by Fukuda Denshi and Nihon Kohden

The QT interval and RR interval in healthy Japanese were measured from 12-lead digital ECGs based on automated algorithms. Fukuda Denshi and Nihon Kohden provided anonymous data sets. Nihon Kohden ECGs were taken at 2010 by ECG-1450. Fukuda Denshi ECGs were taken at 1999 by FCP-4000 series (Algorithm to determine the T wave end is the same as current ECGs.).

**Figure 1:**
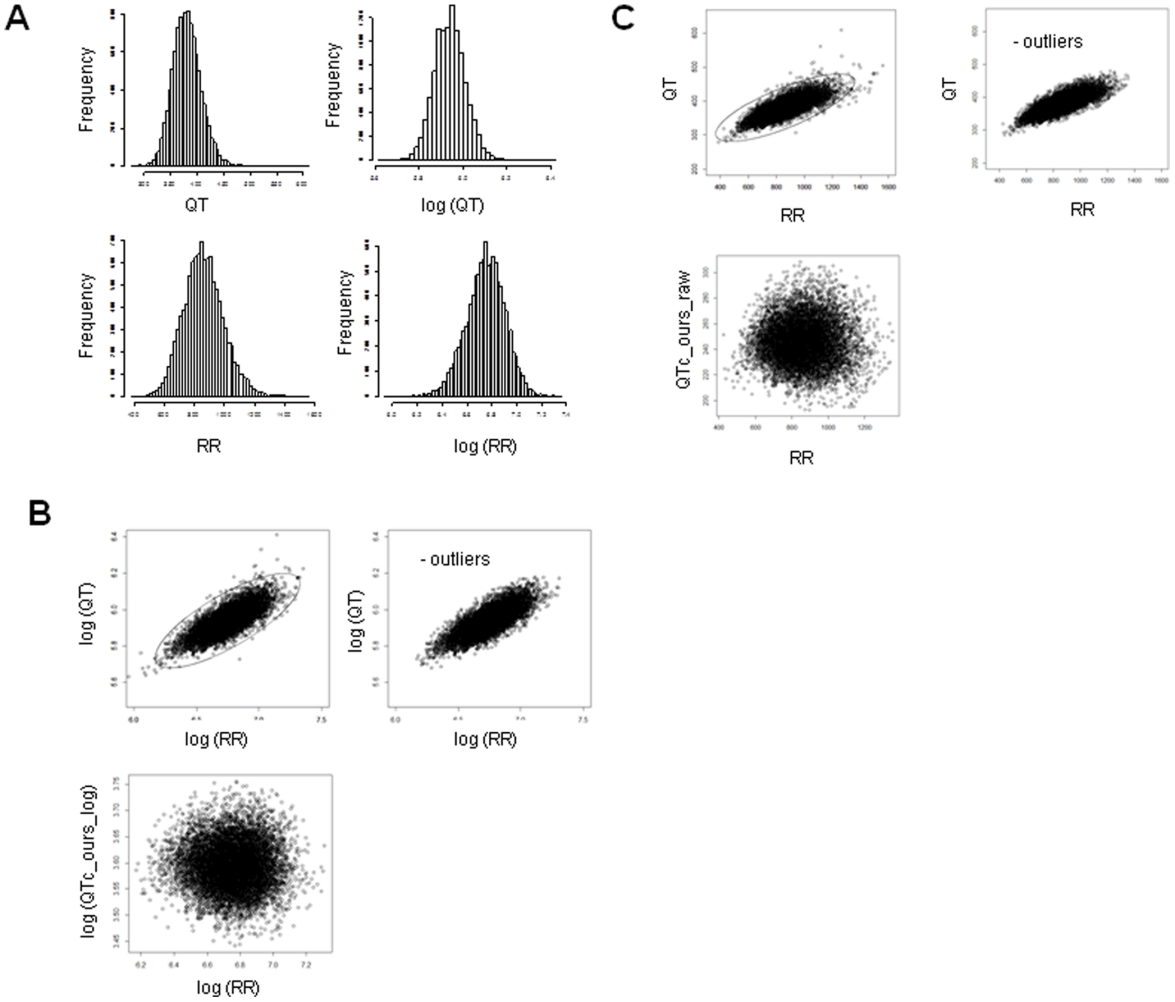
Analysis of resting Fukuda Denshi ECGs. (A) Histograms of QT, log-transformed QT, RR, and log-transformed RR intervals. (B) Scatter plots of log QT versus log RR and log QTc_ours log versus log RR. (C) QT versus RR and QTc_ours raw versus RR. Units of all variables are ms.

**Figure 2:**
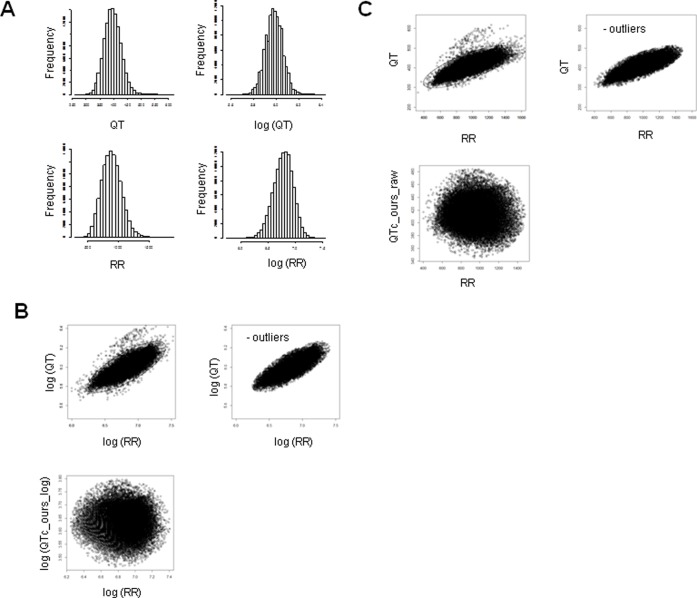
Analysis of resting Nihon Kohden ECGs. (A) Histograms of QT, log-transformed QT, RR, and log-transformed RR intervals. (B) Scatter plots of log QT versus log RR and log QTc_ours log versus log RR. (C) QT versus RR and QTc_ours raw versus RR. Units of all variables are ms.

We evaluated resting ECGs recorded on a Fukuda Denshi machine from 10,529 adult (≥20 years old) healthy subjects (8,631 males aged 46.1±9.2 years and 1,898 females aged 45.3±7.9 years) and resting ECGs recorded on a Nihon Kohden machine from 72,754 adult (≥20 years old) healthy subjects (42,673 males aged 49.9±15.3 years and 30,081 females aged 53.6±16.5 years). Histograms of QT, RR, log-transformed QT, and log-transformed RR intervals demonstrated normal (Gaussian) distributions for these variables (Figure 1A and Figure 2A), although the distribution of each log-transformed variable was closer than the raw variable to the normal distribution (Data S1).

### Definition of outliers

Scatter plots of log QT versus log RR are shown in Figure 1B and Figure 2B. Outliers were defined as the plots outside the contour line that includes 99.9% of the integral of the probability density function of the bivariate normal distribution defined by the means, SDs, and the correlation coefficient of the observed values (log QT versus log RR or QT versus RR) (Figure 1B, 1C, 2B, 2C). The contour line of a bivariate normal distribution is expressed by an ellipse on a plane defined by two means, two SDs and a correlation coefficient. After the ourliers ourside of the ellipse were excluded, the remaining data were used for the analysis.

### Correction of QT by heart rate

Regression analysis was performed separately for the data from two manufacturers, Nihon Koden and Fukuda Denshi for the calculation of optimal heart rate correction formula. In one analysis, we used the log-transformed RR value as an independent variable and the log-transformed QT value as the dependent variable, while in the other, we used raw RR interval as an independent variable and raw QT value as the dependent variable. After performing the linear regression analysis, we used the estimated coefficients for constructing the correction formula. When both independent and dependent variables are in the raw form, the formula used for the regression analysis was as follows.
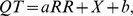
where *a* and *b* are coefficients to be estimated, and X denotes a variable for the variation other than RR that affects QT. Units of QT and RR are sec. Using the estimated coefficients, the formula for the calculation of QTc (ours_raw) was constructed as follows,



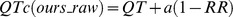
where, *a* is the estimated coefficient.

Note that QTc (ours_raw) = X+*b*+*a*, and this adjustment for the intercept was made so that QTc (ours_raw) = QT when RR = 1.

When both independent and dependent variables are in the log form, the formula used for the regression analysis was as follows.
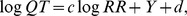
where *c* and *d* are coefficients to be estimated, and Y denotes a variable for the variation other than log RR that affects QT. Using the estimated coefficients, the formula for the calculation of log QTc was constructed as follows,



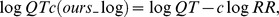



or
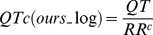



Note that log QTc (ours_log) = Y+*d*, and this adjustment for the intercept was made so that QTc (ours_log) = QT when RR = 1.

We also validated other published heart correction formula as comparison.

The formulas for the correction of QT by Fredericia, Bazzet, Framingham and ECAPs12 methods were as follows.
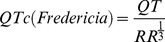





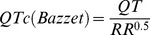





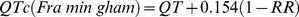
(16)




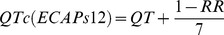



Note that, in all the correction methods, QTc = QT when RR = 1. We calculated those published QTc values using the data from the two manufacturers, and tested for differences between the manufacturers by Student t-test for each gender for each published method for QTc calculation.

### Manufacturer-related effects on QT variation

The values obtained by each correction method for QT were compared between the two manufacturers for each gender by Student t-test.

Next, to examine whether the manufacturer-related differences in automatic measurement of QT interval was independent of other factors such as gender and age, we combined the data sets from Fukuda Denshi and Nihon Kohden ECGs, and performed a multivariate regression analysis. Thus, the combined data were analyzed by the linear multivariate regression model shown by the following formula
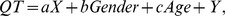
where X denotes a manufacturer (0 or 1), Gender denotes a gender (coded as 1 or 2), Age denotes an age in year and Y denotes the residual variable. We tested whether *a* is equal to 0. Age was expressed by year because relation between QT and age was nearly linear in each gender as judged by the visual inspection.

### Statistical analysis

All statistical analyses were performed using R environment (version 2.15.0). Data are presented as means ± SD. A value of *P*<0.05 was considered statistically significant.

## Results

### Optimal correction formula for QT interval

After the elimination of outliers, the remaining 8,595 male and 1,889 female adult resting Fukuda Denshi ECGs and the remaining 42,398 male and 29,936 female adult resting Nihon Kohden ECGs were subjected to QT interval analysis. In the following formulas for the correction of QT by RR, the units of QT and RR are sec.

Regression analysis using the log-transformed RR value as an independent variable and the log-transformed QT value as the dependent variable produced a regression coefficient of 0.347 (95% CI, 0.342 to 0.352, *P*<2.0×10^−16^, R-squared = 0.609) for Fukuda Denshi ECGs, whereas 0.347 (95%CI, 0.345 to 0.349; *P*<2.0×10^−16^, R-squared = 0.612) for Nihon Kohden ECGs. Therefore, an appropriate correction formula for both Fukuda Denshi and Nihon Kohden would be in the form of QTc (ours_log) = QT×RR^−0.347^. When this correction was made, as expected, the effect of heart rate on QTc was negligible as shown by scatter plots of log (QTc) (ours_log) versus log (RR) (Figure 1B and Figure 2B).

We next tested other published methods as to whether those methods efficiently remove the effects of RR. We performed the linear regression analysis by using log-transformed RR as an independent variable and log-transformed QTc calculated according to Fredericia or Bazzet method as the dependent variable. Note that both Fredericia and Bazzet methods assume the linearity between log-transformed QT and RR. The effect of RR on QTc (Fredericia) remained as shown by the linear regression model using log-transformed variables (β = 0.0103; 95% CI, 0.00513 to 0.0155; *P* = 9.54×10^−5^ for Fukuda Denshi ECGs, β = 0.0144; 95% CI, 0.0125 to 0.0164; *P* = 2×10^−16^ for Nihon Kohden ECGs). If the effect of RR is completely removed, the correlation coefficient β would be 0. The effect of RR on QTc (Bazett) was large as shown by the linear regression model using log-transformed variables (β = −0.156; 95% CI, −0.151 to −0.157; *P* = 2×10^−16^ for Fukuda Denshi ECGs, β = −0.152; 95% CI, −0.150 to −0.154; *P* = 2×10^−16^ for Nihon Kohden ECGs). The regression coefficient after correction by Bazett’s formula indicated excessive correction of QT by RR in normal Japanese resting ECGs, as previously reported [17].

We also performed a regression analysis using raw RR interval as an independent variable and a raw QT value as the dependent variable. After excluding the outliers, the remaining 8,578 male and 1,885 female Fukuda Denshi ECGs and the remaining 42,309 male and 29,960 female Nihon Kohden ECGs were subjected to analysis, which produced a regression coefficient of 0.156 (95%CI 0.154^−^0.158, *P*<2.0×10^−16^, R-squared = 0.608) for Fukuda Denshi ECGs and 0.152 (95%CI, 0.151 to 0.153, *P*<2.0×10^−16^, R-squared = 0.607) for Nihon Kohden ECGs. Therefore, an appropriate correction formula is in the form of QTc (ours_raw) = QT+0.156×(1-RR) for Fukuda Denshi and QTc (ours_raw) = QT+0.152×(1-RR) for Nihon Kohden. When this correction was made, as expected, the effect of heart rate on QTc was negligible as shown by scatter plots of QTc (ours_raw) versus RR (Figure 1C and 2C). This formula is relatively close to the Framingham method, i.e., QTc = QT+0.154×(1-RR).

### Comparison of automatic QT measurements in adult resting ECGs between Fukuda Denshi and Nihon Kohden

Table 1 summarizes our comparison data. The proportion of outliers under the assumption of bivariate normal distribution of log-transformed QT vs. log-transformed RR was larger in Nihon Kohden ECGs than in Fukuda Denshi ECGs, especially in male recordings (0.64% vs. 0.42% in males, *P* = 0.014, by Fisher’s exact test; 0.48% vs. 0.47% in females, *P* = 1.00, by Fisher’s exact test).

**Table 1:** Comparison of automatic QT measurements in adult resting ECGs between Fukuda Denshi and Nihon Kohden.

gendernumberage	QT	RR	QTc (log)		QTc (raw)	
			study	statistic	study	statistic
Nihon Kohden						
			corrected coefficient: 0.347		corrected coefficient: 0.152	
male42,67349.9±15.3	404.0±28.4	947.6±151.2	Ours	412.9±17.8	Ours	412.3±17.4
			Fredericia	412.5±17.8	Framingham	412.2±17.4
			Bazett	417.2±20.8	ECAPs12	411.6±17.4
female30,08153.6±16.5	406.4±27.3	922.6±133.4	Ours	418.8±17.4	Ours	418.4±17.1
			Fredericia	418.3±17.5	Framingham	418.2±17.1
			Bazett	424.8±19.6	ECAPs12	417.4±17.2
Fukuda Denshi						
			corrected coefficient: 0.347		corrected coefficient: 0.156	
male8,63146.1±9.2	378.6±25.7	859.7±132.8	Ours	400.1±16.8	Ours	400.5±15.9
			Fredericia	399.2±17.7	Framingham	400.2±15.9
			Bazett	410.3±20.0	ECAPs12	398.6±15.9
female1,89845.3±7.9	388.1±26.2	876.5±123.3	Ours	407.2±17.7	Ours	407.3±16.8
			Fredericia	406.4±17.7	Framingham	407.0±16.9
			Bazett	416.1±19.7	ECAPs12	405.6±17.0

Units of RR, QT and all corrected forms of QT in this table are ms.

The results of the tests of differences in QT, RR, QTc(ours_log), Fredericia, Bazett, QTc(ours_raw), Framingham and ECAPs12 between Nihon Koden and Fukuda Denshi for each gender were all P<2.2×10^−16^ (Student t-test).

The average uncorrected automatic QT interval was 378.6±25.7 ms in males and 388.1±26.2 ms in females for Fukuda Denshi and 404.0±28.4 ms in males and 406.4±27.3 ms in females for Nihon Kohden. Therefore, the average uncorrected automatic QT interval was longer in Nihon Kohden ECGs than in Fukuda Denshi ECGs (difference of mean; 25.4 ms in males and 18.3 ms in females, *P*<2.2×10^−16^ for both males and females, Student t-test).

After optimizing the heart rate correction of QT interval by the linear regression model using the log-transformed data, QTc (ours_log) interval by Fukuda Denshi in males was 400.1±16.8 ms, and in females was 407.2±17.7 ms, while QTc (ours_log) interval by Nihon Kohden in males became 412.9±17.8 ms, and in females became 418.8±17.4 ms (*P*<2.2×10^−16^ for both males and females, Student t-test). After optimizing the heart rate correction of QT interval by the linear regression model using raw data, QTc (ours_raw) interval by Fukuda Denshi in males was 400.5±15.9 ms, and in females was 407.3±16.8 ms, while QTc (ours_raw) interval by Nihon Kohden in males was 412.3±17.4 ms, and in females was 418.4±17.1 ms (difference between two manufacturers, *P*<2.2×10^−16^ for both males and females, Student t-test). Thus, after optimizing for heart rate correction of QT interval by the linear regression model using either log-transformed data or raw data, the QTc (our method) interval was ∼10 ms longer in Nihon Kohden than Fukuda Denshi ECGs.

### The effect of ECG manufacturer on automatic measurement of QT interval

To examine whether the manufacturer-related differences in automatic measurement of QT interval was independent of other factors such as gender and age, we combined the data sets from Fukuda Denshi and Nihon Kohden ECGs, and performed a multivariate regression analysis using a log-transformed RR value, gender, age and ECG manufacturer as independent variables, and a log-transformed QT value as the dependent variable, as described in the Methods. The analysis produced the following regression coefficients: 0.348 for log-transformed RR (95%CI, 0.346 to 0.349; *P*<2.0×10^−16^), −0.0123 for gender (male) (95%CI, −0.0117 to −0.0129, *P*<2.0×10^−16^), 0.000686 for age (95%CI, 0.000667 to 0.000704, *P*<2.0×10^−16^), and, of note, 0.0275 for ECG manufacturer (Nihon Kohden) (95%CI, 0.0266 to 0.0283, *P*<2.0×10^−16^). Assuming that the average QT interval is 400 ms, the inter-manufacturer difference in QT interval was 11.1 ms.

Similarly, a multivariate regression analysis using raw RR, gender, age and ECG manufacturer as independent variables, and a raw QT value as the dependent variable revealed a regression coefficient for RR of 0.153 (95%CI, 0.152 to 0.153, *P*<2.0×10^−16^), −5.154 for gender (male) (95%CI, −4.917 to −5.391, P<2.0×10^−16^), 0.276 for age (95%CI, 0.268 to 0.284, *P*<2.0×10^−16^), and, of note, 10.610 for ECG manufacturers (Nihon Kohden) (95%CI, 9.808 to 10.962, *P*<2.0×10^−16^), resulting in an inter-manufacturer difference for QT of 10.6 ms.

### Effect of the difference in ECG manufacturer on automatic measurement of RR interval

The automatic RR interval by Fukuda Denshi was 859.7±132.8 ms in males and 876.5±123.3 ms in females, while the automatic RR interval by Nihon Kohden was 947.6±151.2 ms in males and 922.6±133.4 ms in females. We verified the causes of inter-manufacturer differences in RR interval. A multivariate regression analysis using age and ECG manufacturer as independent variables and an RR value as the dependent variable revealed a regression coefficient for ECG manufacturer (Nihon Kohden) of 84.95 in males and 44.72 in females. Surprisingly, RR interval was longer in Nihon Kohden ECGs than in Fukuda Denshi ECGs, even after adjustment for age. Thus, RR interval was 9.43% longer in males and 4.97% longer in females on Nihon Kohden ECGs than on Fukuda Denshi ECGs (*P*<2.0×10^−16^).

## Discussion

What we expect from automatic measurements of QT/QTc interval varies with the times. The automatic approach has been widely used to rule out high-risk patients for SCD in clinical practice. However, as automatic QT measurement technologies have evolved with concomitant increases in precision, automatic measurement becomes a preference, and indeed a necessity, when analyzing 1000 s of ECGs in the setting of either large, multicenter, genome-wide association studies (GWAS) of QT interval identifying common genetic variants among ethnic groups or drug-inducing QT prolongation screening [15]. Automatic QT measurement has advantages over manual measurements from tracings in such settings in terms of reproducibility and time reduction. According to our experience, errors often occur in automatic measurements because the identification of the end of the T wave is difficult in some cases. They were excluded as outliers outside the contour line that includes 99.9% of the integral of the probability density function of the bivariate normal distribution (log QT vs. log RR or raw QT vs. raw RR).

We initiated the present study because the difference in QTc between different manufacturers was big enough to disturb our GWAS for ECG parameters even if it is too little to be noticed in clinical situations. ECG machines from Fukuda Denshi and Nihon Kohden dominate the market of ECG in Japan; however, these companies independently developed automated computer algorithms for QT measurement. In addition, they apply different methods for heart rate correction of QT interval, with Fukuda Denshi machines using Bazett’s formula and Nihon Kohden using the ECAPs12 formula. Despite such key differences, the validity of QT correction formulae and the differences in absolute values of QTc between Fukuda Denshi and Nihon Kohden recordings have never been considered.

Using automatic measurements of QT and RR intervals from 10,529 Fukuda Denshi ECGs and 72,754 Nihon Kohden ECGs recorded for healthy individuals, we found that corrected QTc values calculated by either Bazett’s or ECAPs12 formulae had residual heart rate dependence after correction. When we used log-transformed data, the optimal heart rate correction formula of QT interval was equivalent between Fukuda Denshi and Nihon Kohden systems, and would be in the form of QTc (our method) = QT×RR^−0.347^. However, when we used raw data, the optimal heart rate correction formula of QT interval was in the form of QTc (our method) = QT+0.156×(1-RR) for Fukuda Denshi and QTc (our method) = QT+0.152×(1-RR) for Nihon Kohden. Since our study included the largest number of subjects from the Japanese ancestry, we proposed our own correction formulas for QT to adjust for RR.

In addition, after optimization of heart rate (HR) correction of QT interval by the linear regression model using either log-transformed data or raw data, QTc (our method) interval was ∼10 ms longer in Nihon Kohden ECGs than in those recorded on Fukuda Denshi machines. Indeed, regression analysis revealed that differences in the ECG machine used had up to a two-fold larger impact on QT variation than gender difference. Such an impact is likely to be of considerable importance when ECGs for a given individual are recorded on different machines in the setting of multi-institutional joint research. For example, the effect of various factors (gene, gender, age, drug etc) on QT interval can never be evaluated without the information about the ECG manufacturer.

The explanation for this difference remains speculative but one would emphasize that Fukuda Denshi and Nihon Kohden adopt different definitions for measurement of QT interval. Fukuda Denshi adopts the average duration of QT interval, in which QT interval is defined as the mean of 12-lead measurements. By contrast, Nihon Kohden adopts the global duration of QT interval, in which QT interval is defined by the earliest QRS onset in one lead and latest offset of T-end in any other lead (wave onset and offset do not necessarily appear at the same time in all leads because the activation of wave fronts propagates differently). Such difference should yield longer QT interval by Nihon Kohden ECG machine than Fukuda Denshi ECG machine.

Surprisingly, even after adjustment for age, the automatic measurements of RR interval are 9.43% longer in males and 4.97% longer in females on Nihon Kohden ECGS than on those recorded by Fukuda Denshi systems. So far, we have no explanation for such a big difference in RR interval, which Fukuda Denshi calculates as the mean RR interval during recording time, whereas Nihon Kohden calculates RR interval by length of time between the first beat and the last beat during recording time divided by number of beats −1. Neither manufacturer excludes premature beats from automatic measurements of RR interval.

In conclusion, inter-manufacturer difference of QT interval was ∼10 ms even after optimizing the heart rate correction of QT interval. Based on these study results, we recommend that ECG machines of the same manufacturer are used to measure QT and RR intervals in the setting of multi-institutional joint research. It is desirable to unify the computer algorithm for automatic QT and RR measurements from an ECG and optimize the heart rate correction formula of QT interval. Otherwise, interchangeable formula should be established.

### Limitations

A motive of the present study was that we found clear differences in average QTc's between different sampling sites for 3,000 healthy individuals. Although they were clustered into two different groups, we were able to identify neither the manufacturers nor the correction formula for QTc in each sampling site. In contrast, the manufacturers and correction formula but not phenotypes are clear in the present data set with about 80,000 subjects. Therefore, a limitation of this study is that the two different data sets are quite different in nature since the former is from healthy individuals while phenotypes of the latter are unknown, the manufacturer data are obtained from the latter but not from the former and the correction formula are obtained from the latter but not from the former.

There are, in general, two different approaches in medicine, i.e. one by the analysis of a small number of individuals in detail and the other by analyzing big data from multiple individuals using statistics. Both of the approaches are important because each of them has drawbacks and both of the approaches compensate for the drawbacks of each other. Our present study employed the latter approach.

This is a retrospective study. Since we were not able to collect all covariates including BMI, some factors not included in our analysis may have been important confounding factors. So far, we have no explanation for a difference in RR interval between ECG machine manufacturers. We could not exclude the confounding variables that influence autonomic nervous system activity.

To the best of our knowledge, this is the first work to assess the outcomes with different automated measurements of QT interval between ECG machine manufacturers. The next step is to obtain ECG data from many individuals for each of whom the machines from the two manufacturers are used at the same time.

## Supporting Information

Data S1 **The log-transformed QT and RR assumed a closer to normal distribution than the raw variables.**
(DOCX)

## References

[1] Tomaselli GF, Beuckelmann DJ, Calkins HG, Berger RD, Kessler PD, et al (1994) Sudden cardiac death in heart failure. The role of abnormal repolarization. Circulation 90: 2534–2539. 7955213 10.1161/01.cir.90.5.2534

[2] Straus SM, Kors JA, De Bruin ML, van der Hooft CS, Hofman A, et al (2006) Prolonged QTc interval and risk of sudden cardiac death in a population of older adults. J Am Coll Cardiol 47: 362–367. 16412861 10.1016/j.jacc.2005.08.067

[3] Vrtovec B, Delgado R, Zewail A, Thomas CD, Richartz BM, et al (2003) Prolonged QTc interval and high B-type natriuretic peptide levels together predict mortality in patients with advanced heart failure. Circulation 107: 1764–1769. 12665499 10.1161/01.CIR.0000057980.84624.95

[4] Schouten EG, Dekker JM, Meppelink P, Kok FJ, Vandenbroucke JP, et al (1991) QT interval prolongation predicts cardiovascular mortality in an apparently healthy population. Circulation 84: 1516–1523. 1914093 10.1161/01.cir.84.4.1516

[5] Salvi V, Karnad DR, Panicker GK, Kothari S (2010) Update on the evaluation of a new drug for effects on cardiac repolarization in humans: issues in early drug development. Br J Pharmacol 159: 34–48. 19775279 10.1111/j.1476-5381.2009.00427.x PMC2823350

[6] Splawski I, Shen J, Timothy KW, Lehmann MH, Priori S, et al (2000) Spectrum of mutations in long-QT syndrome genes. KVLQT1, HERG, SCN5A, KCNE1, and KCNE2. Circulation 102: 1178–1185. 10973849 10.1161/01.cir.102.10.1178

[7] Priori SG, Schwartz PJ, Napolitano C, Bloise R, Ronchetti E, et al (2003) Risk stratification in the long-QT syndrome. N Engl J Med 348: 1866–1874. 12736279 10.1056/NEJMoa022147

[8] Moss AJ, Schwartz PJ, Crampton RS, Tzivoni D, Locati EH, et al (1991) The long QT syndrome. Prospective longitudinal study of 328 families. Circulation 84: 1136–1144. 1884444 10.1161/01.cir.84.3.1136

[9] Newton-Cheh C, Eijgelsheim M, Rice KM, de Bakker PI, Yin X, et al (2009) Common variants at ten loci influence QT interval duration in the QTGEN Study. Nat Genet 41: 399–406. 19305408 10.1038/ng.364 PMC2701449

[10] Pfeufer A, Sanna S, Arking DE, Muller M, Gateva V, et al (2009) Common variants at ten loci modulate the QT interval duration in the QTSCD Study. Nat Genet 41: 407–414. 19305409 10.1038/ng.362 PMC2976045

[11] Roden DM (2006) Long QT syndrome: reduced repolarization reserve and the genetic link. J Intern Med 259: 59–69. 16336514 10.1111/j.1365-2796.2005.01589.x

[12] Noseworthy PA, Havulinna AS, Porthan K, Lahtinen AM, Jula A, et al (2011) Common genetic variants, QT interval, and sudden cardiac death in a Finnish population-based study. Circ Cardiovasc Genet 4: 305–311. 21511878 10.1161/CIRCGENETICS.110.959049 PMC3119024

[13] Darpo B, Agin M, Kazierad DJ, Layton G, Muirhead G, et al (2006) Man versus machine: is there an optimal method for QT measurements in thorough QT studies? J Clin Pharmacol 46: 598–612. 16707406 10.1177/0091270006286900

[14] Hnatkova K, Gang Y, Batchvarov VN, Malik M (2006) Precision of QT interval measurement by advanced electrocardiographic equipment. Pacing Clin Electrophysiol 29: 1277–1284. 17100684 10.1111/j.1540-8159.2006.00532.x

[15] Couderc JP, Garnett C, Li M, Handzel R, McNitt S, et al (2011) Highly automated QT measurement techniques in 7 thorough QT studies implemented under ICH E14 guidelines. Ann Noninvasive Electrocardiol 16: 13–24. 21251129 10.1111/j.1542-474X.2010.00402.x PMC3076006

[16] Sagie A, Larson MG, Goldberg RJ, Bengtson JR, Levy D (1992) An improved method for adjusting the QT interval for heart rate (the Framingham Heart Study). Am J Cardiol 70: 797–801. 1519533 10.1016/0002-9149(92)90562-d

[17] Funck-Brentano C, Jaillon P (1993) Rate-corrected QT interval: techniques and limitations. Am J Cardiol 72: 17B–22B. 10.1016/0002-9149(93)90035-b 8256750

