# Supplementary material for: Evaluation of Differences in Automated QT/QTc Measurements between Fukuda Denshi and Nihon Koden Systems
Source: PLoS One. 2014 Sep 17;9(9):e106947. doi: 10.1371/journal.pone.0106947 (PMC4167700; doi:10.1371/journal.pone.0106947)
Supplement: Data S1 — The log-transformed QT and RR assumed a closer to normal distribution than the raw variables. (DOCX) [file pone.0106947.s001.docx]

**Data S1**

**The log-transformed QT and RR assumed a closer to normal distribution than the raw variables**

**Fukuda Denshi:**

After excluding outliers of raw and log-transformed values of QT and RR, we examined which variables more closely fit a normal distribution. Observation of both histograms and QQ plots suggested that log-transformed QT rather than raw QT more closely assumed a normal distribution (data not shown). In addition, examination of the moments around the means indicated that both skewness (0.071) and kurtosis (3.044) of log QT more closely assumed a normal distribution (0 and 3) than skewness (0.224) and kurtosis (3.128) of raw QT values. However, observation of both histograms and QQ plots did not clarify which variable, raw or log-transformed RR, more closely assumed a normal distribution. Examination of the moments around the means also provided no conclusive results because skewness and kurtosis of log RR were -0.178 and 3.12, respectively, while those of raw RR were 0.205 and 3.06, respectively.

**Nohon Kohden**

After excluding outliers of raw and log-transformed values of QT and RR, we observed from both histograms and QQ plots that log-transformed QT rather than raw QT more closely assumed a normal distribution (data not shown). In addition, examination of the moments around the means indicated that both skewness (-0.012) and kurtosis (3.079) of log QT more closely assumed a normal distribution (0 and 3) than skewness (0.148) and kurtosis (3.130) of the raw QT values. For the RR values, neither histograms nor QQ plots indicated which variable, raw or log-transformed, more closely assumed a normal distribution. Examination of the moments around the means also failed to provide conclusive results because skewness and kurtosis of log RR were -0.195 and 3.23, respectively, while those of raw RR were 0.206 and 3.16, respectively.
